# Rare Truncating *HAND2* Variants Predispose to Atrial Fibrillation

**DOI:** 10.3390/genes17060701

**Published:** 2026-06-17

**Authors:** Hong Zhang, Xiao-Qing Hu, Ning Li, Dao-Liang Zhang, Xing-Biao Qiu, Chen-Xi Yang, Ying-Jia Xu, Yi-Qing Yang, Juan Wang

**Affiliations:** 1 Department of Obstetrics, Shanghai East Hospital, Tongji University School of Medicine, Shanghai 200123, China; zhanghong_2025@163.com; 2 General Practice of Western Medicine, Community Health Service Center of Gonghexin Road Sub-District, Jing’an District, Shanghai 200072, China; huxiaoqingyeye@163.com; 3 Department of Cardiology, The First Affiliated Hospital of Shenzhen University, Shenzhen 518035, China; frankleelx@163.com; 4 Cardiac Arrhythmia Center, Fuwai Hospital, Chinese Academy of Medical Sciences, Shenzhen 518057, China; xkzhangdaoliang@126.com; 5 Department of Cardiology, Shanghai Chest Hospital, Shanghai Jiao Tong University School of Medicine, Shanghai 200030, China; qiuxingbiao@hotmail.com; 6 Department of Cardiology, Shanghai Fifth People′s Hospital, Fudan University, Shanghai 200240, China; cxyang21@m.fudan.edu.cn; 7 Department of Geriatrics, Huashan Hospital, Fudan University, Shanghai 200040, China; xuyingjia@5thhospital.com; 8 Department of Cardiology, Longhua Hospital, Shanghai University of Traditional Chinese Medicine, Shanghai 200032, China

**Keywords:** arrhythmia, atrial fibrillation, human genetics, HAND2, biochemical assay

## Abstract

**Background/Objectives**: As the most prevalent supraventricular arrhythmia, affecting around 1% of people worldwide, atrial fibrillation (AF) is implicated with a multitude of detrimental clinical sequelae, encompassing congestive failure, thromboembolic cerebral stroke, and premature death. Accumulating epidemiological evidence convincingly demonstrates genetic defects as a cornerstone in the pathogenesis of idiopathic AF. Despite significant genetic underpinnings responsible for AF, owing to substantial genetic heterogeneity, the predisposing genetic substrates for AF in most individuals remain to be ascertained. **Methods**: A four-generation pedigree suffering from familial AF and a cohort of 238 subjects affected with idiopathic AF, together with 266 healthy people, were enrolled prospectively. Pan-exome sequencing analysis was implemented on the chosen AF pedigree members, and Sanger sequencing assay was performed on all research subjects. The functional effects of the detected *HAND2* variations were examined via an in vitro dual-reporter gene measurement. **Results**: Two novel heterozygous truncating *HAND2* variations, NM_021973.3: c.138G>A; p.(Trp46*) and NM_021973.3: c.337C>T; p.(Gln113*), were discovered in the AF pedigree and one of the 238 AF cases, respectively. The two *HAND2* variants co-segregated with the AF phenotype and were absent from the 532 control chromosomes of 266 healthy individuals. Quantitative reporter gene assays unveiled that both Trp46*- and Gln113*-mutant HAND2 failed to transcriptionally activate *HCN4* and *NPPA*, two AF-causative genes. Additionally, the two variations nullified the synergistic transcriptional activation of *NPPA* by HAND2 and GATA4, another recognized gene predisposing to AF. **Conclusions**: These findings support *HAND2* as a strong candidate gene contributing to AF susceptibility, which unravels novel etiopathogenesis underpinning the occurrence and perpetuation of AF and offers a potential molecular target for individualized medicine.

## 1. Introduction

Atrial fibrillation (AF) symbolizes the most prevalent type of clinical cardiac arrhythmia, impacting roughly 1% of people globally [1,2]. Notably, in real-world scenarios, approximately one third of AF patients are asymptomatic and are not diagnosed until the first cardiovascular/cerebral event occurs; therefore, the true prevalence of AF is obviously underestimated [3,4]. Several epidemiological investigations suggested that asymptomatic AF comprised about 40% of all already-diagnosed AF, and this figure was anticipated to keep rising [5]. A retrospective cohort study showed that in the United States, around 11% of AF cases were undiagnosed [6], and a recent epidemiological survey of silent AF revealed that the real prevalence of AF in the general population was at least 3% if instrument-detected asymptomatic AF was included [7]. Hence, AF has become the third most common heart disease, after arterial hypertension and ischemic heart disease [8].

In addition to decreased health-related quality of life [9,10,11] and physical exercise endurance [12,13,14], AF may also contribute to a great variety of other adverse clinical outcomes, including atrial thrombosis [15,16,17]; systemic thromboembolism encompassing thromboembolic/ischemic cerebral stroke [18,19], hemorrhagic stroke [20,21,22], cognitive function decline, and early-onset dementia [23,24,25]; acute kidney damage as well as chronic renal disease [26,27]; acute myocardial infarction [28,29,30]; atrial cardiomyopathy [31,32,33,34]; tachycardia-induced cardiomyopathy [35,36,37]; congestive cardiac failure [38,39,40,41]; life-threatening ventricular arrhythmias and cardiac arrest [42]; and even early-onset cardiac death [43,44,45,46]. AF is responsible for a substantially increased healthcare resource utilization/healthcare disbursement, with a mean total healthcare expenditure of USD 63,031 for each patient per year, USD 27,896 greater than those with no AF [47,48]. Clearly, AF has contributed to substantial morbidity and mortality and imposed an enormous economic encumbrance [1,47,48,49]. Despite great importance, the etiopathogenesis of AF remains uncertain in the majority of subjects.

It is increasingly recognized that the etiologies underlying AF are extremely intricate and diverse, and that both non-inherited precipitating factors and genetic abnormalities can lead to the genesis and perpetuation of AF [1,2,50,51,52,53]. Emerging work points to a significant role of non-heritable risk factors in the pathogenesis of AF, such as unhealthy lifestyle, obesity, cardiac surgery, obstructive sleep apnea, myocardial infarction, diabetes mellitus, primary hypertension, hyperthyroidism, hypertrophic/dilated cardiomyopathy, bacterial/viral myocarditis, gut microbiota dysbiosis, pulmonary embolism, and exposure to toxicants/air pollutants [50,51,54,55,56,57,58,59,60,61,62]. However, emerging strong evidence underscores the pivotal roles of inherited components in the development and sustainment of AF [2,52,53]. To date, over 60 AF-causative genes have been uncovered, of which most code for voltage-gated K^+^ channel sub-units (such as K^+^ channel α-subunits KCNH2 and KCNQ1; K^+^ channel β-subunits KCNE1 and KCNE2), hyperpolarization-activated potassium channels (such as HCN4), Na^+^ channel subunits (such as α-sub-unit KCNA5; β-subunits SCN1B and SCN4B), Ca^2+^ channel subunits (such as Ca^2+^ channel β2-sub-unit CACNB2; Ca^2+^ channel α2δ4-subunit CACNA2D4), gap junction channel proteins (such as GJA5/Cx40 and GJA1/Cx43), myocardial constitutive proteins (such as titin/TTN), signal molecules (such as atrial natriuretic peptide precursor A/NPPA), and transcription factors (such as short-stature homeobox 2/SHOX2 and GATA-binding protein 4/GATA4) [2,52,53,63,64,65,66,67]. Over the past decade, massive genome comparison studies between AF cases and healthy control individuals in large populations have facilitated the discovery of single-nucleotide variations in more than 520 genomic loci related to AF, encompassing the genetic loci at chromosomes 1q21 (KCNN3), 16q22 (ZFHX3), and 4q25 (PITX2), though the biological functions are still unclear for the overwhelming majority of these variants linked to AF [2,52,53,68,69,70]. Nevertheless, due to the striking genetic heterogeneity, the genetic components vulnerable to AF in a larger proportion of cases remain to be determined [2,52,53,63,64,65,66,67,68,69,70]. Consequently, the current study aimed to identify and characterize a new gene linked to AF susceptibility.

## 2. Materials and Methods

### 2.1. Research Individuals

For this research program, a four-generation family with AF (arbitrarily named Family-112AF) and another group of 238 AF-affected patients, along with 266 healthy volunteers who had no AF history, were enlisted from the Han-ethnicity Chinese population. Diagnosis of AF was definitely made by expert cardiologists, utilizing the data from electrocardiograms in terms of the AHA/ACC/HRS guideline for AF [71]. The criteria used for the inclusion and exclusion of AF cases have been consistently described elsewhere [63,67]. Clinical categorization of AF was performed as elaborated previously [71]. Phenotypic information was collected from each study participant, including familial/medical history, physical examination results, electrocardiograms, and echocardiograms, along with regular laboratory test findings. A 24 or 48 h ambulatory electrocardiogram/Holter was conducted when indicated. The controls were age- and sex-matched, and electrocardiographic screening was performed in all controls to actively exclude subclinical AF. The current research protocol gained approval from the ethical review committee of Shanghai Fifth People’s Hospital Affiliated to Fudan University (approval number: 2022-179; approval time: 23 October 2024), and the research conformed strictly to the tenets of the Helsinki Declaration. All research participants or their legal guardians offered informed consent prior to their recruitment. Peripheral blood specimens were collected from all participating individuals, from which genomic DNA was routinely isolated.

### 2.2. Genetic Analysis of Research Participants

Whole-exome sequencing (WES), along with a bioinformatics assay, was implemented in nine AF-affected members and ten non-AF members from Family-112AF, as described previously [63,67,72,73,74]. In brief, 5 µg of purified genomic DNA from each participant selected for WES were fragmented to construct an exome DNA library and captured using the SureSelect Human All Exon (V6) Kit (Agilent Technologies, Santa Clara, CA, USA). Sequencing of exome libraries was performed under the HiSeq 4000 Genome Analyzer (Illumina, San Diego, CA, USA). Raw data produced by WES were processed using the Illumina Analysis Pipeline (v2.6; Illumina), and alignments of obtained sequencing reads to the human reference genome (hg19/GRCh37) were performed with the BWA software (v2.10.12). Sequence variations, encompassing single-nucleotide variations as well as small insertion/deletion variations, were identified by exploiting the Genome Analysis Toolkit (GATK) software (version 3.8.1.0) and annotated with ANNOVAR (v2018). Variants were further filtered based on their frequency in the population genetics databases of gnomAD (v4.1.1), 1000 Genomes, ExAC, and dbSNP (v151); their predicted functional effect; and their inheritance mode. Firstly, variants with a minor allele frequency exceeding 0.01% in population genetics databases were filtered out. Secondly, variants that did not show autosomal-dominant inheritance were filtered out. Thirdly, synonymous, intronic, and intergenic variants were excluded, while the variants possibly affecting functional consequences were kept (missense, nonsense, splicing site, frameshift, and non-frameshift insertion/deletion variants). Finally, the rare non-synonymous variants that were predicted in silico to be pathogenic by several algorithms (MutationTaster, PolyPhen-2, PROVEAN, LRT, and SpliceAI) were kept. Variants were further classified according to the guidelines of the American College of Medical Genetics and Genomics and the Association for Molecular Pathology (ACMG/AMP) [75,76]. Sanger sequencing analysis was performed on the genomic DNAs of all family members available from Family-112AF to confirm a candidate AF-related variant and to ascertain the co-segregation of a rare pathogenic variation with the AF phenotype in the family (Family-112AF). A Sanger sequencing examination of the coding regions, in conjunction with the splicing boundaries, of the gene containing a discovered AF-related nucleotide mutation was made in the second population of 238 AF patients, along with 266 unrelated non-AF healthy subjects, who served as controls. As narrated elsewhere [67], the databases of dbSNP (updated 11 March 2025) and gnomAD (v4.1.1), which were accessed on 16 December 2025, were retrieved to ascertain whether the observed AF-related variations were new.

### 2.3. Construction of Gene-Expressing Vectors

The preparation of human cardiac cDNAs has been described elsewhere [72]. Utilizing the prepared cDNA as a template, a 805 bp DNA fragment containing the whole coding region of the wild-type *HAND2* (heart and neural crest derivatives expressed 2) gene (NM_021973.3) was amplified via polymerase chain reaction (PCR) employing a hot-start DNA polymerase kit (Cat.# 14966001; Invitrogen/ThermoFisher Scientific, Carlsbad, CA, USA) and the *HAND2*-specific primer pair of 5′-TACGAATTCGACGCCGAGCGCTGAGGCAG-3′ and 5′-TACGTCGACGTCTGCATCTGGCGCCTTGG-3′. The amplicons/*HAND2* cDNAs were fragmented by gel electrophoresis and purified using a gel purification kit (Cat.# 20051; Qiagen GmbH, Hilden, Germany). The extracted human *HAND2* cDNA and the pCI-neo vector (Cat.# E1841; Promega, Madison, WI, USA) were digested with the restrictive enzymes of *EcoR*I and *Sal*I (Cat.# ER0271 and Cat.# ER0641, respectively; Thermo Scientific, Waltham, MA, USA), fragmented by agarose gel electrophoresis, isolated using a gel extraction kit (Cat.# 20051; Qiagen GmbH, Hilden, Germany) and ligated by using the T_4_ DNA ligase to produce the wild-type HAND2-pCI-neo vector. Using the wild-type HAND2-pCI-neo as a PCR template, the Trp46*-mutant HAND2-pCI-neo vector was produced via site-directed mutagenesis by exploiting a site-targeted mutagenesis kit (Cat.# A13282; Invitrogen/ThermoFisher Scientific, Carlsbad, CA, USA) and the primers of 5′-CTACTTCCATGGCTGACTCATCGGCCACCCC-3′ and 5′-GGGGTGGCCGATGAGTCAGCCATGGAAGTAG-3′. Similarly, the vector of Gln113*-mutant HAND2-pCI-neo was created through site-directed mutagenesis utilizing the primer pair of 5′-GAGCGGCGCAGGACTTAGAGCATCAACAGCG-3′ and 5′-CGCTGTTGATGCTCTAAGTCCTGCGCCGCTC-3′. In addition, a 683 bp promoter fragment (from −2817 to −2135, with the transcription start nucleotide numbered +1) of the human *HCN4* gene (NC_000015.10) harboring an HCN4-binding motif (5′-CAGATG-3′) was produced by PCR amplification from human genomic DNA using a hot-start DNA polymerase kit (Cat.# 14966001; Invitrogen/ThermoFisher Scientific, Carlsbad, CA, USA) and the *HCN4*-specific primers of 5′-GTAGCTAGCGAGACCCAGGTGGAGCAATG-3′ and 5′-GTACTCGAGATCTGGCTTCTCCTGCC-3′. The amplified human *HCN4* promoter DNA fragment was inserted into the reporter gene vector of pGL3-Basic (Cat.# E1751; Promega, Madison, WI, USA) at the *Nhe*I-*Xho*I sites to create the HCN4-luciferase (HCN4-luc) reporter gene vector, where the human *HCN4* promoter drives the expression of firefly luciferase. The GATA4-pcDNA3.1(+) expression vector, which expresses wild-type human GATA4, and the NPPA-luciferase (NPPA-luc) reporter gene vector, where the human *NPPA* promoter drives the expression of firefly luciferase, were constructed as previously elaborated [77]. Each of the aforementioned vectors was validated via DNA sequencing assay.

### 2.4. Cellular Culture and Transfection with Vectors as Well as Dual-Reporter Assay

As described elsewhere [72], HeLa cells were cultured for 36 h, and then transfection with the indicated expression vectors was performed, utilizing a lipofectamine transfection reagent (Cat.# 15338030; Invitrogen; Waltham, MA, USA). Specifically, cells were transfected with 0.2 µg of empty pCI-neo vector, 0.2 µg of wild-type human HAND2-pCI-neo vector, 0.2 µg of Trp46*-mutant human HAND2-pCI-neo vector, 0.2 µg of Gln113*-mutant human HAND2-pCI-neo vector, 0.1 µg of wild-type human HAND2-pCI-neo vector + 0.1 µg of empty pCI-neo vector, 0.1 µg of wild-type human HAND2-pCI-neo vector + 0.1 µg of Trp46*-mutant human HAND2-pCI-neo vector, or 0.1 µg of wild-type human HAND2-pCI-neo vector + 0.1 µg of Gln113*-mutant human HAND2-pCI-neo vector, together with 0.8 µg of HCN4-luc vector and 2 ng of the pGL4.75 vector expressing Renilla luciferase (Cat.# E6931; Promega, Madison, WI, USA). To evaluate the synergistic transactivation, HeLa cells were transfected with 0.2 µg of each expression vector (empty pCI-neo vector, wild-type human HAND2-pCI-neo vector, Trp46*- or Gln113*-mutant human HAND2-pCI-neo vector, wild-type human GATA4-pcDNA3.1(+) vector, wild-type HAND2-pCI-neo vector + wild-type GATA4-pcDNA3.1(+), Trp46*-mutant HAND2-pCI-neo + wild-type GATA4-pcDNA3.1(+), Gln113*-mutant HAND2-pCI-neo + wild-type GATA4-pcDNA3.1(+) vector), along with 1.0 μg of NPPA-luc and 2 ng of pGL4.75 (Cat.# E6931; Promega, Madison, WI, USA). As described elsewhere [72], the HeLa cells were collected, and the dual-luciferase activities were successively analyzed by exploiting a dual-reporter assay kit (Cat.# E2920; Promega, Madison, WI, USA). The activities of the *HCN4* and *NPPA* promoters were shown as the ratios of firefly to Renilla luminescence intensities. For every expression vector applied to the present functional analysis, three parallel transfection experiments were conducted in triplicate.

### 2.5. Statistics

Statistical assessment was completed by employing the SPSS software (version 17.0; SPSS, New York, NY, USA). As described elsewhere [72], the comparisons of continuous parameters, which were normally distributed and presented as mean ± standard deviation (SD), were conducted by leveraging the Student’s *t*-test for pairwise comparisons, while ANOVA (analysis of variance) along with the Tukey–Kramer test were used for multi-group comparisons. The comparison of categorical data, expressed as counts along with percentages, was implemented between two groups with the χ^2^ test or Fisher test. A two-sided *p* < 0.05 indicated a significant difference.

## 3. Results

### 3.1. Phenotypic Features of the Research Individuals

As exhibited in Figure 1, one four-generation, thirty-three-member family suffering from AF (Family-112AF) was identified, which consisted of thirty living and three dead family members. All individuals from Family-112AF underwent electrocardiography and echocardiography, and two individuals (IV-4 and IV-7) from Family-112AF also underwent Holter monitoring.

In Family-112AF, eleven family members, including eight female and three male members, were definitely diagnosed with AF based on their electrocardiographic findings/medical proceedings. Other than congenital heart disease (affecting four family members), no other known non-genetic risk factors predisposing to AF were observed in the individuals from Family-112AF, such as hypertension, valvular disease, thyroid disease, sleep apnea, excessive alcohol intake, obesity, cardiomyopathy, inflammatory disease, pulmonary disease, or prior cardiac surgery. The proband (individual III-6 from Family-112AF), a female member aged 43 years with a 12-year AF history, was admitted to the hospital because of an acute recurrent attack of syncope. Her representative electrocardiogram showing AF is presented in Figure 2.

The proband (individual III-6 from Family-112AF) successfully had her AF terminated by trans-catheter radiofrequency ablation during her stay in the hospital. Both her father (individual II-1 from Family-112AF) and grandfather (individual I-1 from Family-112AF) had over 30 years of AF history and succumbed to an ischemic cerebral stroke. The proband’s elder sister (individual III-2 from Family-112AF), younger brother (individual III-7 from Family-112AF), and uncle (individual II-3 from Family-112AF), along with her uncle’s younger daughter (individual III-12 from Family-112AF) and son (individual III-13 from Family-112AF), also experienced a successful ablation treatment of AF. The proband’s other relatives affected with AF, encompassing individuals IV-1, IV-4, and IV-7 from Family-112AF, did not accept surgical/trans-catheter interventional treatment for AF until the current recruitment but had taken antiarrhythmic drugs for AF. The proband’s unaffected relatives, including 17 female and 16 male individuals, had negative histories of AF episodes, with their 12-lead electrocardiograms showing proper sinus rhythm. The phenotypic characteristic profiles from the living pedigree members diagnosed with AF are summarized in Table 1.

In addition, four (II-3, III-12, III-13, and IV-7 from Family-112AF) of the nine members alive with AF also had congenital heart disease, namely, congenital ventricular septal defect.

Moreover, another group of 238 patients with idiopathic AF experienced a comprehensive clinical assessment, in comparison with 266 unrelated healthy subjects with a negative AF history, who were enrolled as controls. The demographic and phenotypic characteristics of this group of AF patients, as well as the control individuals, are outlined in Table 2.

### 3.2. Detection of Two New HAND2 Variations Predisposing to AF

WES and informatics assay were implemented in nine AF-affected members (individuals II-3, III-13, III-12, III-7, III-6, III-2, IV-7, IV-4, and IV-1) and ten healthy members (individuals II-6, II-4, II-2, III-11, III-10, III-5, III-1, IV-8, IV-5, and IV-2) from Family-112AF (Figure 1), generating an average of 23 Gb of DNA sequence data per individual sample, with a mean of 97% of DNA sequences mapped to the human genome (hg19) and 76% to the target sequences. The average sequencing depth was ~200X. A mean of 16,972 rare variants (range 16,105–18,604) per participant member passed inheritance model filtering, of which only one heterozygous nonsense variant—chr4: 174,450,303C>T (GRCh37.p13 Primary Assembly/hg19: NC_000004.11), equal to chr4: 173,527,168G>A (GRCh38.p14 Primary Assembly/hg38: NC_000004.12) or NM_021973.3: c.138G>A; p.(Trp46*), which passed pathogenicity filtering—was shared by the nine affected family members and absent in the ten unaffected family members. This was confirmed by Sanger sequencing analysis to co-segregate with the AF phenotype in the whole pedigree (Family-112AF), with a maximum two-point logarithm of odds (LOD) score of 2.6015 at a recombination fraction (θ) of 0.05. As displayed in Figure 3, the sequencing electropherograms illustrate the heterozygous c.138G>A variation in *HAND2*, together with its wild-type DNA control.

Moreover, Sanger sequencing analyses of the coding sequences together with splicing junctions of *HAND2* were implemented in all participants by employing the primers given in Table 3, which verified that the mutation of NM_021973.3: c.138G>A; p.(Trp46*) in *HAND2* was carried by all affected individuals but by none of the healthy individuals of Family-112AF (as given in Table 1). There were no unaffected carriers, age-dependent penetrance, or untested family members who could affect interpretation. Genetic examination of Family-112AF indicated an autosomal-dominant transmission of AF in the entire family.

In addition, sequence screening of the entire coding exons together with splicing donors/acceptors of *HAND2* in another cohort of 238 patients with AF revealed a heterozygous *HAND2* variation of NM_021973.3: c.337C>T; p.(Gln113*) in a male 30-year-old case, who had no family history of AF or congenital heart disease. In the *HAND2* c.337C>T variation carrier, paroxysmal AF was first diagnosed by electrocardiogram at the age of 27 years and treated with anti-arrhythmia drugs, which developed into long-standing persistent AF and was terminated by catheter-based radiofrequency ablation at the age of 30 years. His echocardiographic findings were normal, with no structural heart disease. This *HAND2* variant was not observed in his parents, whose electrocardiograms and echocardiograms were normal during follow-up, with no AF or structural heart disease or stroke history, suggesting a de novo *HAND2* variation with unlikely parental mosaicism. The sequence chromatogram images displaying the c.337C>T mutation in *HAND2*, alongside its wild-type DNA control, are depicted in Figure 4.

A standard electrocardiogram documenting AF recorded from the patient carrying the *HAND2* c.337C>T mutation is presented in Figure 5.

Neither of the two *HAND2* variations (c.138G>A and c.337C>T) implicated with AF was observed in the 266 non-AF persons, nor in the genetics databases of gnomAD (accessed on 16 December 2025) nor dbSNP (accessed on 16 December 2025), supporting that the two *HAND2* variations were novel. The two variants in *HAND2* were both classified as pathogenic mutations according to the ACMG/AMP criteria.

### 3.3. Inability of Trp46*- or Gln113*-Mutant HAND2 to Transcriptionally Activate HCN4

As Figure 6A demonstrates, in the HeLa cells transfected with needed vectors, including empty pCI-neo vector (−), wild-type human HAND2-pCI-neo vector (HAND2), and Trp46*-mutant human HAND2-pCI-neo vector (Trp46*), separately or in combination, HAND2 and Trp46* transactivated *HCN4* by ~15-fold and ~1-fold, respectively (HAND2 vs. Trp46*: t = 11.2175; *p* = 0.0004). When HAND2 and Trp46* were co-expressed, the induced transactivation of *HCN4* was ~7-fold (HAND2 vs. Trp46* + HAND2: t = 5.8044; *p* = 0.0044), while the induced transactivation of *HCN4* by empty vector (−) and Trp46* was ~8-fold (HAND2 + empty vector vs. Trp46* + HAND2: t = 1.5882; *p* = 0.1874), indicating no dominant-negative effect. Additionally, similar statistical outcomes were obtained from multiple comparisons (F = 78.8084, *p* = 1.607 × 10^−7^). Specifically, for HAND2 vs. Trp46*, t = 13.6167 and *p* < 0.0001; for HAND2 vs. HAND2 + Trp46*, t = 7.43 and *p* < 0.0001; and for HAND2 + (−) vs. HAND2 + Trp46*, t = 1.17 and *p* = 0.7063. In concert, as Figure 6B exhibits, HAND2 and Gln113* transactivated *HCN4* by ~14-fold and ~1-fold, respectively (HAND2 vs. Gln113*: t = 10.5235; *p* = 0.0085). When HAND2 and Gln113* were co-expressed, the induced transcriptional activation of *HCN4* was ~7-fold (HAND2 vs. Gln113* + HAND2: t = 5.4522; *p* = 0.0055), while the induced transactivation of *HCN4* by empty vector (−) and Gln113* was ~8-fold (HAND2 + empty vector vs. Gln113* + HAND2: t = 1.5721; *p* = 0.1910), indicating no dominant-negative effect. In addition, comparable statistical outcomes were generated from multiple comparisons (F = 70.9991, *p* = 2.654 × 10^−7^). Specifically, for HAND2 vs. Gln113*, t = 12.5667 and *p* < 0.0001; for HAND2 vs. HAND2 + Gln113*, t = 6.82 and *p* = 0.0001; and for HAND2 + (−) vs. HAND2 + Gln113*, t = 1.0467 and *p* = 0.7585.

### 3.4. Failure of Trp46*- or Gln113*-Mutant HAND2 to Transcriptionally Activate NPPA Alone or in Synergy with GATA4

As delineated in Figure 7A, in the HeLa cells transfected with indicated expression vectors, including empty pCI-neo vector (−), wild-type human HAND2-pCI-neo vector (HAND2), Trp46*-mutant human HAND2-pCI-neo vector (Trp46*), and GATA4-pcDNA3.1(+) vector (GATA4), singly or together, HAND2 and Trp46* transactivated *NPPA* by ~6-fold and ~1-fold, respectively (HAND2 vs. Trp46*: t = 10.9797; *p* = 0.0004). In combination with GATA4, HAND2 and Trp46* transactivated *NPPA* by ~35-fold and ~14-fold, respectively (HAND2 + GATA4 vs. Trp46* + GATA4: t = 9.4254; *p* = 0.0007). In addition, similar statistical outcomes were yielded from multiple comparisons (F = 164.0029, *p* = 1.295 × 10^−10^). Specifically, for HAND2 vs. Trp46*, t = 5.2933 and *p* = 0.0242; and for HAND2 + GATA4 vs. Trp46* + GATA4, t = 20.2467 and *p* < 0.0001. Analogously, as given in Figure 7B, HAND2 and Gln113* transactivated *NPPA* by ~6-fold and ~1-fold, respectively (HAND2 vs. Gln113*: t = 12.606; *p* = 0.0002). In combination with GATA4, HAND2 and Gln113* transactivated *NPPA* by ~33-fold and ~13-fold, respectively (HAND2 + GATA4 vs. Gln113* + GATA4: t = 8.1668; *p* = 0.0012). In addition, comparable statistical outcomes were attained by multiple comparisons (F = 124.5007, *p* = 6.547 × 10^−10^). Specifically, for HAND2 vs. Gln113*, t = 5.4233 and *p* = 0.0339; and for HAND2 + GATA4 vs. Gln113* + GATA4, t = 19.3733 and *p* < 0.0001.

## 4. Discussion

In the current human genetics research, two new heterozygous truncating *HAND2* variations, NM_021973.3: c.138G>A; p.(Trp46*) and NM_021973.3: c.337C>T; p.(Gln113*), were unmasked in a four-generation pedigree affected with familial AF and in one of two hundred and thirty-eight cases suffering from idiopathic AF, respectively. The two *HAND2* variations, which were absent from 532 human reference chromosomes, co-segregated with AF in the families. Quantitative dual-luciferase measurements of the two *HAND2* mutations unveiled that both Trp46*- and Gln113*-mutant HAND2 failed to transcriptionally activate *HCN4* and *NPPA*, two AF-causing genes [64,78,79,80,81,82,83,84]. Furthermore, the two mutations diminished the synergistic transactivation of *NPPA* by HAND2 and GATA4, another gene causative for AF [85,86,87]. Therefore, genetically compromised *HAND2* may contribute to AF by reducing the expression of its downstream genes, encompassing *HCN4* and *NPPA*. Notably, although segregation analysis supported autosomal-dominant inheritance, age-dependent penetrance and variable expressivity cannot be excluded.

To date, two members of the Twist family of transcription factors harboring a conserved basic helix–loop–helix (bHLH) domain have been discovered, including HAND2 and HAND1, which are involved in a network of transcription factors underlying cardiovascular development [88]. The HAND factors may form hetero- or homodimers with their bHLH-containing partners and bind to the consensus DNA sequences, the so-called D-box (5′-CGNNTG-3′) or E-box (5′-CANNTG-3′), within the target gene promoters, functioning to mediate the expression of downstream genes [88]. Lineage-tracing expression studies have ascertained the different and overlapping expression profiles for the two HAND genes in the developing heart, encompassing endocardium, myocardium, epicardium, and neural crest cells [88]. *HAND2* is located on human chromosome 4q34.1, which encodes a bHLH-containing protein consisting of 217 amino acids [89]. The expression of HAND2 was first detectable within the cardiac and endocardial progenitors in the cardiac crescent of mouse embryo, and the robust expression of HAND2 was observed within the pharyngeal mesoderm of the second heart field that contributes to the developing heart tube [88]. Through cardiac looping, endocardial expression of HAND2 was most robust, with low-level myocardial expression of HAND2 observed [88]. In addition, the expression of HAND2 has also been observed in the cardiac neural crest cells, the myocardial cuff within the outflow tract, and the proepicardial organ forming epicardium [88]. In humans, the high-level expression of HAND2 has been determined in both embryonic and adult hearts [90,91,92]. It has been substantiated that HAND2 mediates the expression of multiple genes, including *HCN4*, *TBX3*, *TBX18*, and *NKX2.5*, in cooperation with GATA4, TBX5, and MEF2C, and plays a crucial role in coordinating the formation of pacemaker-like myocytes [93]. Notably, in addition to *HCN4* and *GATA4*, variations in *NKX2.5* and *TBX5* have also been identified to give rise to AF [94,95,96,97]. Moreover, a battery of desmosome genes is specifically activated in induced pacemaker-like myocytes in the presence of HAND2 through coordinated chromatin re-organization, and desmosomes have been demonstrated to exert a key role in coordinating the assembly as well as the function of gap junction channels, which are key to intercellular electrical coupling [93,98]. Furthermore, HAND2 has been validated to directly regulate the expression of *NPPA,* independent of its DNA-binding affinity [99], and *NPPA* mutations have been found to cause AF [64,82,83,84]. Collectively, these results support *HAND2* loss-of-function variations as a molecular mechanism underpinning AF.

It may also be attributed to atrial electrophysiological abnormalities that *HAND2* dysfunction predisposes to AF, in addition to abnormal atrial development and structural remodeling, such as atrial fibrosis caused by reduced expression of NPPA [100,101,102,103], which may be at least in part due to the observed dysfunctional *HAND2*. It has been demonstrated that atrial structural remodeling, encompassing extracellular matrix expansion between cardiomyocytes and fibrosis, impairs electrical coupling between adjacent cardiomyocytes, slows conduction, and interferes with electrical continuity, forming a substrate vulnerable to AF [100,101]. The observed loss of HAND2-mediated HCN4 transcriptional activation is particularly noteworthy because HCN4 is essential for cardiac pacemaker activity and sinoatrial node function. Dysfunction of the sinoatrial node and atrial conduction system may facilitate atrial arrhythmogenesis through electrical instability, abnormal automaticity, and atrial remodeling [78,79,80,81].

In humans, genome-wide association studies (GWAS) have revealed that a chromosome 4q34.1 locus harboring *HAND2* is associated with enhanced susceptibility to AF in several populations of different countries [104,105,106]. Low et al. [104] performed a GWAS in 8180 AF patients and 28,612 non-AF control persons, with follow-up/replication in an additional 3120 AF patients and 125,064 non-AF control persons. As a result, sixteen chromosomal loci exceeding the threshold of genome-wide significance were identified, of which seven were previously associated with AF, encompassing loci at 16q22 (*ZFHX3*), 12q24 (*TBX5* and *CUX2*), 1q24 (*PRRX1*), 10q24 (*NEURL1*), 6q22 (*GJA1*–*HSF2*), 7q31 (*CAV1*), and 4q25 (*PITX2*); nine were newly associated with AF, encompassing loci at 10q24 (*SH3PXD2A*), 4q34 (*HAND2* as well as 3′ of *HAND2*), 1q32 (*PPFIA4*), 9q22 (*SYK*), 5q31 (*HBEGF*), 1p13 (*KCND3*), 2p14 (*SLC1A4*–*CEP68*–*RAB1A*), and 10q12 (*NEBL*) [100]. In the replication stage, only six of the nine newly identified loci were specifically related to AF in the Japanese population, including rs7698692 at 3′ of *HAND2*, rs2296610 in *NEBL*, rs12044963 in *KCND3*, rs2540953 between *SLC1A4* and *CEP68*, rs2047036 in *SH3PXD2A*, and rs17461925 in *PPFIA4* [104]. Although no significant association of rs17059534 in *HAND2* with AF was determined in the second stage, the combined *p* value of 3.38 × 10^−10^ from the meta-analysis implied that this SNP was suggestively associated with AF [104]. Lee et al. [105] carried out a GWAS in 672 patients with AF and 3700 control individuals with no AF and a replication study in 200 independent AF cases and 1812 non-AF controls. Resultantly, five previously recognized genomic loci (16q22/*ZFHX3*, 1q24/*PRRX1*, 12q24/*TBX5,* 10q24/*NEURL*, and 4q25/*PITX2*) were verified, and two new genomic loci were correlated to early-onset AF, including 4q34.1/*HAND2* (*rs8180252*, close to *HAND2* encoding long non-coding RNA, *p* = 1.49 × 10^−11^) and 1q32.1/*PPFIA4* (*rs11579055*), which were replicated in an additional independent cohort of samples in stage 2 [105]. In addition, Okubo et al. [106] scanned 540 AF cases and 520 non-AF people for genomic variations previously associated with AF by GWAS and discovered five genomic variations (in or near *HAND2*, *PITX2*, *ZFHX3*, *NEURL1*, and *PRRX1*) associated with AF following Bonferroni correction. These observational results support that genetically defective *HAND* predisposes to AF.

Interestingly, in the present investigation, four members (II-3, III-12, III-13, and IV-7 from Family-112AF) of the nine members alive with AF also had congenital heart disease, namely, congenital ventricular septal defect. In fact, multiple *HAND2* mutations have been previously uncovered to result in an array of congenital heart diseases, encompassing ventricular/atrial septal defect, endocardial cushion/atrioventricular septal defect, tetralogy of Fallot, pulmonic/aortic stenosis, pulmonary atresia, patent ductus arteriosus, double-outlet right ventricle, and unicuspid aortic valve [107,108,109,110,111]. These results highlight the pivotal role of *HAND2* in the proper embryonic development of the cardiovascular system and indicate that *HAND2* variants are more commonly associated with congenital heart disease than isolated AF. The phenotypic differences associated with *HAND2* variations may be explained, at least in part, by distinct genetic backgrounds, incomplete penetrance, and age-dependent expressivity. In addition, provided that AF is well recognized as a long-term sequela of congenital septal defects due to chronic atrial volume overload, structural remodeling, and post-surgical changes, independent of any primary arrhythmia gene, the coexistence of AF and congenital septal defects in several mutation carriers raises the possibility that cardiac structural abnormalities may contribute to the arrhythmogenic substrate associated with or independent of *HAND2* mutations. Considering HAND2 is a cardiac developmental transcription factor, we could not rule out the possibility that the observed AF could be secondary to congenital structural heart disease, atrial remodeling, surgical/interventional treatment, altered hemodynamics, or a shared developmental substrate rather than a direct AF phenotype. Nevertheless, the AF phenotype in VSD-carrying mutation carriers could be attributed primarily to the *HAND2* variant itself rather than to structural heart disease, because it occurred early, and in the same family, most members carrying the *HAND2* variation had only early-onset AF without congenital heart disease.

The crucial roles of *HAND2* in embryonic cardiogenesis and post-natal structural remodeling have been elucidated in animals [112,113,114,115,116,117,118,119]. During murine cardiogenesis, HAND2 is expressed predominantly in the right ventricles, and targeted knockout of *Hand2* in murine embryos leads to embryonic demise due to hypoplastic right ventricular, vascular defects, and heart failure [112]. In mice, HAND2 is also expressed highly in the mesenchyme, which underlies the branchial arch epithelium, and homozygous knockout of *Hand2* leads to hypoplasia of the first and second arches, secondary to programmed cell death and failure to form the third and fourth arches [113]. Morikawa et al. [114,115] reported that in addition to regulating the development of the heart outflow tract and the second heart field, HAND2 also exerted an effect on regulating the development of the sympathetic nervous system [114,115]. Videira et al. [116] reported that cardiac-restricted deletion of *Hand2* led to severe heart dysfunction under right-ventricular pressure overload [116]. George et al. [117] reported that knockout of *Hand2* in the post-natal conduction system of the murine heart resulted in decreased PR interval; deletion of *Hand2* in the left ventricle of the murine embryo led to prolongation of the PR interval as well as enhanced vulnerability to atrial arrhythmias [117]. VanDusen et al. [118] reported that conditional deletion of *Hand2* in murine embryos (loss of *Hand2* in Periostin lineage cells) led to lethality shortly after birth, due to catecholamine deficiency (because of dramatically reduced dopamine β hydroxylase, tyrosine hydroxylase, and phenylethanolamine N-methyltransferase), sinus bradycardia, and subsequent heart failure [118]. Song et al. [119] reported that in vitro, HAND2, TBX5, MEF2C, and GATA4 could cooperatively reprogram adult murine cardiac/tail-tip fibroblasts into beating cardiac-like myocytes; in vivo/in mice, in dividing non-cardiomyocytes, these transcription factors could reprogram these cells into cardiac-like myocytes, improve cardiac function, and reduce adverse ventricular remodeling after myocardial infarction [119]. In addition, Guo et al. [120] reported that *Hand2*-knockout cardiomyocytes derived from human embryonic stem cells had decreased markers of atrial cardiomyocytes and showed ventricular-like action potentials. Moreover, in humans, a loss-of-function *HAND2* variation has been found to predispose to familial dilated cardiomyopathy [89]. Taken collectively, these investigations underscore the essential roles of HAND2 in cardiac organogenesis and post-natal adaptation, especially in regulating the development and sustainment of the heart conduction system and sympathetic nervous system, and strongly suggest that genetically compromised *HAND2* predisposes to arrhythmias and congenital heart diseases.

There are limitations to our studies, especially to the functional studies with HeLa cells. First, the functional assays were performed exclusively in HeLa cells, which are a cervical epithelial cancer line with no cardiac transcriptional architecture, no cardiomyocyte-specific cofactors, and no electrophysiological phenotype. Given that HAND2 is a cardiac developmental transcription factor whose activity is highly context-dependent—requiring interaction with GATA4, TBX5, and MEF2C and cardiac chromatin accessibility—the significance of transactivation data from HeLa cells rather than cardiomyocytes is questionable, mainly because functional explorations performed in HeLa cells rather than a cardiac cell model may not fully recapitulate the transcriptional regulatory environment of human atrial tissue. Functional experiments in a more cardiac-relevant model, such as HL-1 cells, AC16 cells, neonatal cardiomyocytes, or induced pluripotent stem-cell-derived cardiomyocytes (iPSC-CMs), or ideally human atrial cardiomyocytes, would strengthen the functional findings substantially. Nevertheless, HeLa cells have been commonly used to analyze the functional effects of the mutations of cardiac developmental transcription factor genes, such as GATA4 and HAND2 [121,122], in order to avoid the influence of various abundant endogenous transcription factors, which is why HeLa cells were chosen over cardiac alternatives (HL-1, AC16, neonatal rat ventricular cardiomyocytes, or iPSC-CMs). Second, the functional consequences of truncating *HAND2* mutations need further clarification with regard to nonsense-mediated mRNA decay (NMD), protein instability, and abnormal subcellular distribution of the mutant HAND2 proteins. Previous experimental studies have demonstrated that a premature translation termination codon can cause mRNA degradation in organisms and cell lines via a mechanism called NMD, a translation-dependent, multi-step process, which monitors and degrades faulty or irregular mRNAs [123]. In the present study, two nonsense mutations in *HAND2* were predicted to produce two premature translation termination codons; therefore, the mutant *HAND2* mRNAs were likely to undergo NMD, although not all nonsense mutations elicited it [124]. At present, we cannot exclude NMD in these *HAND2* mutation carriers due to the unavailability of their heart samples, where the mutant HAND2 proteins may be expressed. Even though the mutant *HAND2* mRNAs underwent NMD, the overall abundance of *HAND2* mRNAs would be reduced by half, leading to haploinsufficiency, which was consistent with functional outcomes. Notably, the splicing of downstream introns or pre-mRNA—a process essential for depositing the exon–junction complex (EJC), a multiprotein assembly, roughly 20–24 nucleotides upstream of every exon–exon junction—is a prerequisite for the degradation of mRNAs harboring a premature termination codon via the NMD pathway. Consequently, NMD cannot occur in the context of cDNA constructs [123]. Future studies should first determine whether the identified truncating variations elicit NMD of *HAND2* mRNA. Third, although HAND2 has been determined to bind to three evolutionarily conserved E-box consensus sites (cis-elements/promoters residing within a 258 bp proximal region) and regulate the transcription upregulation of the *NPPA* gene [99], alone or synergistically with GATA4 [121] or MEF2C [125,126], whether the promoter regions used in the present reporter assays are physiologically relevant in atrial tissue and direct binding evidence, such as chromatin immunoprecipitation (ChIP) or electrophoretic mobility shift assay (EMSA), remain to be determined; this is also the case for the *HCN4* gene, because its promoter has E-box consensus sites and its expression level has been associated with NPPA [127,128]. Finally, the sporadic AF cohort comprised 238 cases, and 266 controls were employed. For a study claiming to characterize AF susceptibility gene variants in a Chinese Han population, this is a relatively modest sample. While the two *HAND2* variants were absent from the 266 healthy controls, the power to detect differences in the prevalence of this rare variant was limited.

## 5. Conclusions

The current study first links *HAND2* loss-of-function mutations to AF, supporting *HAND2* as a strong candidate gene contributing to AF susceptibility, which offers novel insight into the etiopathogenesis of AF, and provides a molecular target for personalized prophylaxis and treatment of AF.

## Figures and Tables

**Figure 1:**
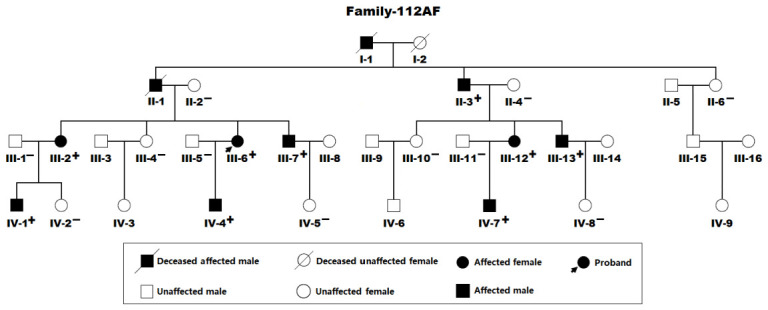
Pedigree with atrial fibrillation. This family was named Family-112AF. Here, “+” marks a member carrying the observed *HAND2* variation; “−” marks a non-carrier for the observed *HAND2* variation.

**Figure 2:**
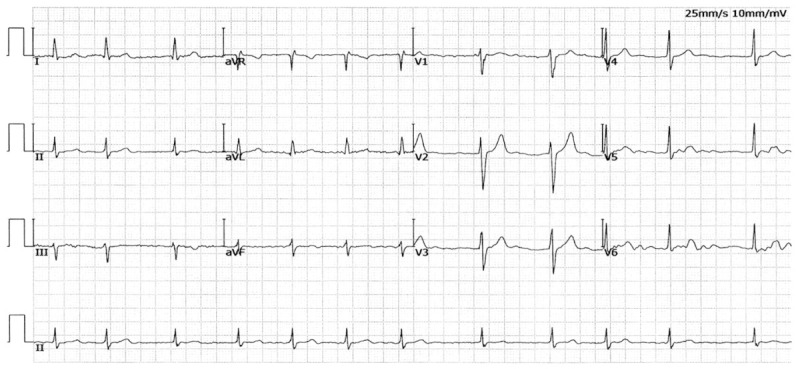
A standard electrocardiogram recorded from the proband (individual III-6 from Family-112AF). The 12-lead electrocardiogram documents the diagnosis of atrial fibrillation.

**Figure 3:**
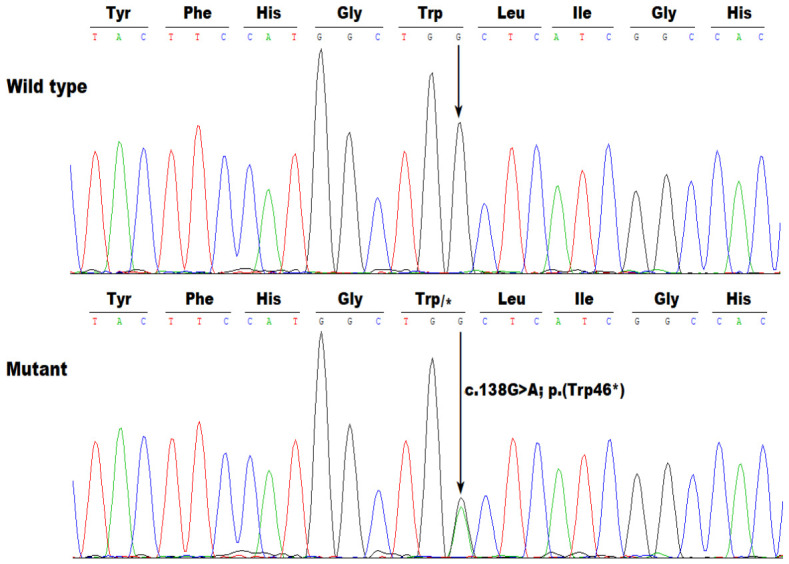
A new *HAND2* mutation linked to atrial fibrillation (AF). Sequence electropherograms illustrating the heterogeneous *HAND2* variation detected in the proband suffering from AF (Mutant), alongside its wild-type DNA sequence from a non-AF individual (Wild type). A vertical arrow orients to the *HAND2* variation.

**Figure 4:**
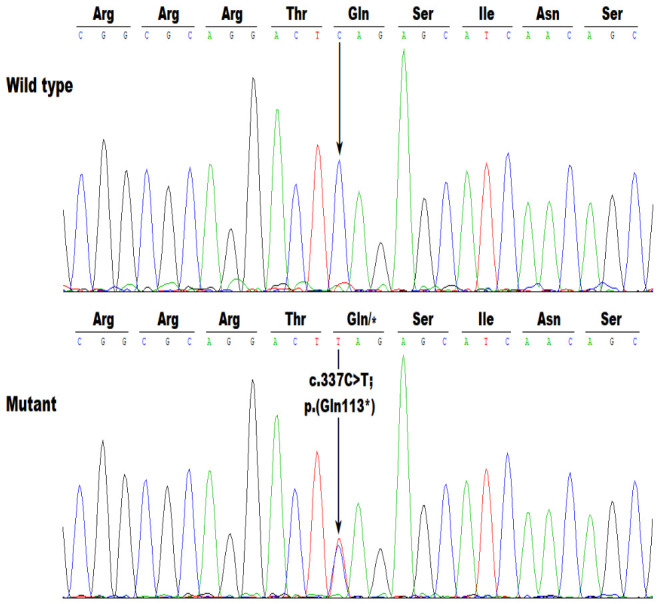
A de novo human *HAND2* variation predisposing to atrial fibrillation (AF). The heterogeneous *HAND2* mutation (Mutant) was discovered in one of two hundred and thirty-eight cases affected with AF, and its wild-type DNA control was found in a non-AF subject (Wild type).

**Figure 5:**
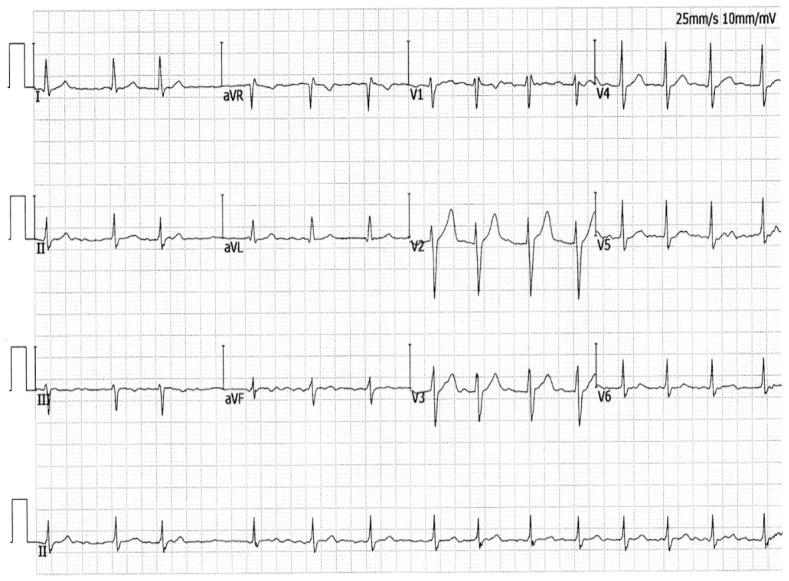
A standard electrocardiogram from the patient with atrial fibrillation (AF) harboring the *HAND2* c.337C>T mutation. This 12-lead electrocardiogram illustrates AF.

**Figure 6:**
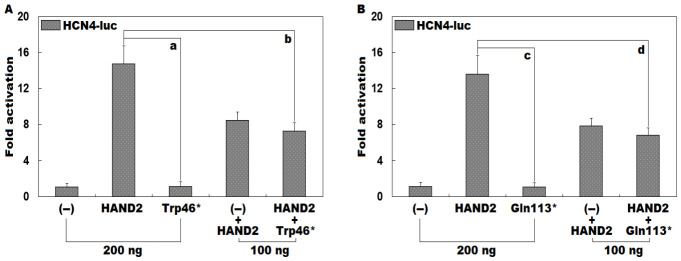
Inability of Trp46*- or Gln113*-mutant HAND2 to transactivate *HCN4*. In HeLa cells, dual-reporter gene assays uncovered that neither the Trp46* mutant (**A**) nor the Gln113* mutant (**B**) was able to transactivate *HCN4*. Here, “a” represents *p* < 0.001; “b”, “c”, and “d” signify *p* < 0.01, in contrast to wild-type HAND2 (200 ng).

**Figure 7:**
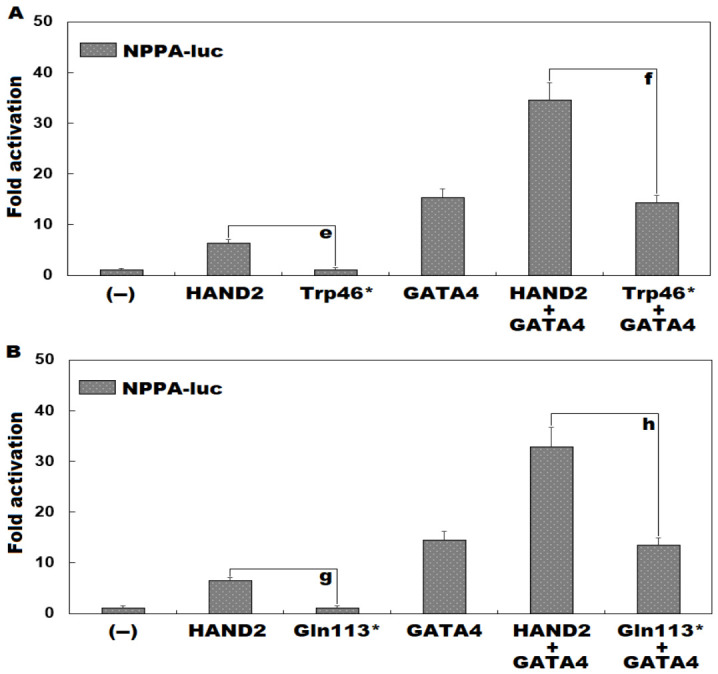
Synergistic transactivation of *NPPA* between HAND2 and GATA4 nullified by the Trp46* or Gln113* mutation. In the HeLa cells expressing indicated vectors, biochemical measurements using dual-reporter genes revealed that the synergistic transactivation of *NPPA* by HAND2 and GATA4 was nullified by the Trp46* mutation (**A**) or the Gln113* mutation (**B**). Here, “e”, “f”, and “g” all denote *p* < 0.001, and “h” means *p* < 0.005, when compared with the wild-type counterparts.

**Table 1:** Phenotypical features of the living family members diagnosed with atrial fibrillation.

Individual Parameter				Heart Phenotype	Electrocardiograph			Echocardiograph		Genotype
Identity (Family-112AF)	Sex	Age at onset of AF (Years)	Age at Evaluation of AF (Years)	AF (Clinical Classification), CHD	Heart Rate (Beats/Min)	QRS Interval (ms)	QTc (ms)	LAD (mm)	LVEF (%)	*HAND2* Variation (c.138G>A)
II-3	M	21	66	LSP, CHD	98	120	441	47	45	+/−
III-2	F	35	47	LSP	84	127	432	39	57	+/−
III-6	F	31	43	LSP	71	111	425	37	61	+/−
III-7	M	37	41	LSP	93	98	416	35	63	+/−
III-12	F	20	42	LSP, CHD	107	116	434	37	60	+/−
III-13	M	23	40	LSP, CHD	79	105	398	34	62	+/−
IV-1	M	26	26	Persistent	86	94	403	32	65	+/−
IV-4	M	21	21	Paroxysmal	75	92	392	29	67	+/−
IV-7	M	20	20	Paroxysmal, CHD	91	95	431	27	64	+/−

LAD, left atrial diameter; F, female; QTc, corrected QT interval; AF, atrial fibrillation; M, male; LVEF, left ventricular ejection fraction; LSP, long-standing persistent; CHD, congenital heart disease; +/−, heterozygote for *HAND2* c.138G>A variation.

**Table 2:** Demographic and phenotypic characteristics of the 238 cases suffering from atrial fibrillation as well as 266 healthy control individuals.

Variable	Patient (n = 238)	Control (n = 266)	*p* Value
Age (years)	57.92 ± 7.61	58.03 ± 8.36	0.8778
Gender (female/male)	112/126	128/138	0.8117
BMI (kg/m^2^)	23.96 ± 3.41	24.03 ± 3.55	0.8220
Family history of AF (%)	62 (26.05)	0 (0)	<0.0001 *
History of prior ischemic cerebral stroke (%)	14 (5.88)	0 (0)	<0.0001 *
History of prior pacemaker implantation (%)	16 (6.72)	0 (0)	<0.0001 *
Fasting plasma glucose (mmol/L)	4.39 ± 0.52	4.41 ± 0.57	0.6821
Total cholesterol (mmol/L)	4.23 ± 0.49	4.26 ± 0.53	0.5113
Triglyceride (mmol/L)	1.40 ± 0.36	1.42 ± 0.38	0.5457
Diastolic BP (mmHg)	85.64 ± 6.51	86.01 ± 7.13	0.5449
Systolic BP (mmHg)	126.69 ± 8.73	127.10 ± 9.06	0.6061
Resting HR (beats/min)	78.15 ± 8.36	77.82 ± 8.24	0.6560
LAD (mm)	38.06 ± 6.41	35.73 ± 4.19	<0.0001 *
Left ventricular ejection fraction (%)	61.84 ± 6.53	62.26 ± 7.02	0.4887

AF, atrial fibrillation; BP, blood pressure; HR, heart rate, BMI, body mass index; LAD, left atrial diameter. * *p* < 0.05.

**Table 3:** Primers designed to amplify the human *HAND2* gene.

Coding Exons	Forward Primers (5′→3′)	Reverse Primers (5′→3′)	Amplicons (bp)
1-part 1	CCGGGAAGCAGGAGCCGAAG	CGCTGTTGATGCTCTGAGTC	687
1-part 2	AGGGCGAAATGAGTCTGGTAG	TGGTGCAAACAACCTTGAAGC	697
2	GAGGCTCCTTCGCCCTATTT	GACGGCTTTTCCGGAGTCCT	575

## Data Availability

The two *HAND2* variations analyzed during this study, including NM_021973.3: c.138G>A; p.(Trp46*) and NM_021973.3: c.337C>T; p.(Gln113*), have been deposited in the LOVD database, with two variant numbers of 0001068177 (https://databases.lovd.nl/shared/variants/0001068177; accessed on 10 February 2026) and 0001068178 (https://databases.lovd.nl/shared/variants/0001068178; accessed on 10 February 2026), respectively. The other datasets analyzed during this study are available from the corresponding author upon reasonable request.
